# Veterinarians’ attitudes and practices regarding opioid-related vet shopping practices in tri-state Appalachian counties: an exploratory study

**DOI:** 10.1186/s12917-020-02428-x

**Published:** 2020-06-22

**Authors:** Falguni C. Patel, Jeffrey A. Raines, Richard W. Kim, Karen Gruszynski, Robert E. Davis, Manoj Sharma, Gilbert Patterson, Jason W. Johnson, Vinayak K. Nahar

**Affiliations:** 1 DeBusk College of Osteopathic Medicine, Lincoln Memorial University, Harrogate, TN USA; 2 Center for Animal and Human Health in Appalachia, College of Veterinary Medicine, Lincoln Memorial University, Harrogate, TN USA; 3 Substance Use and Mental Health Laboratory, Department of Health, Human Performance and Recreation, University of Arkansas, Fayetteville, AR USA; 4 Department of Behavioral & Environmental Health, School of Public Health, Jackson State University, Jackson, MS USA; 5 Department of Dermatology, School of Medicine, University of Mississippi Medical Center, 2500 North State Street – L216, Jackson, MS 39216 USA; 6 Department of Preventive Medicine, School of Medicine/John D. Bower School of Population Health, University of Mississippi Medical Center, Jackson, MS USA

**Keywords:** Vet shopping, Opioid, Veterinarians, Attitudes, Practice, Prescription drug misuse

## Abstract

**Background:**

The opioid crisis continues to grow in the United States with 46,700 drug overdose deaths due to opioids in 2017 alone. Vet shopping, the practice of soliciting veterinarians for prescription medications, has been receiving national media attention in recent years. A 2014 review of Prescription Monitoring Drug Programs found less than 10 veterinary shoppers nationwide. Still much is unknown about the role of vet shopping and the opioid crisis. This study sought to understand the practice of vet shopping through the eyes of veterinarians practicing in Appalachian counties within the states of Kentucky, Tennessee, and Virginia, United States.

**Results:**

Fourteen veterinarians were asked a set of 13 questions related to vet shopping. Results indicated that 13 veterinarians in the study had heard of the phenomenon of vet shopping and eight veterinarians had personally encountered vet shopping in their practices. Qualitative analysis of the interviews identified six key themes including a need for interprofessional communication and how important a valid veterinary-client-patient-relationship is within the profession.

**Conclusions:**

The study hopefully sheds some light on the how often vet shopping is encountered in practice, concerns of veterinarians regarding vet shopping, and potential areas for improvement.

## Background

In the United States (US), the opioid crisis continues to grow. According to the Centers for Disease Control and Prevention (CDC), there were 70,237 Americans who died from a drug overdose in 2017 with 46,700 (67.8%) due to opioids [1]. Of particular interest is the rise and prevalence of opioid addiction in the Appalachian Region, which consists of 420 counties in 13 states in the Eastern United States (US). In two recent CDC reports, several states within Appalachia were shown to have significant increases in deaths related to opioid abuse [1, 2]. The persistence of the opioid epidemic produces many secondary problems including fetal/neonatal opioid dependence, increased transmission of infectious diseases due to intravenous drug use and the inability of convicted drug users to find employment [3]. People often develop tolerance and dependence to opioids after chronic exposure which could be a consequence of a legitimate need for pain mitigation.

With ever changing laws and new criteria for prescribing opioid pain medications, drug seekers have become more creative in their efforts to obtain opioids. One of the newest avenues for drug seeking behavior is veterinary clinics [4]. “Vet shopping” is the solicitation of veterinarians to obtain a controlled substance prescription for their animal with no intent of administering to their animal [5]. Recent media reports have thrusted vet shopping into the spotlight. Within the last several years, a raid of dog breeding operation in Oregon found approximately 100,000 Tramadol pills in the owners’ possession, a Kentucky woman who was arrested for intentionally cutting her dog in order to obtain Tramadol, and a man in Fairfax County, Virginia who was visiting multiple veterinary clinics to obtain prescription medications for his pet [6–9]. It is still unclear what impact vet shopping actually has on the opioid epidemic.

In 2014, a fifty state survey of Prescription Drug Monitoring Programs (PDMP) found that there were less than ten veterinary shoppers identified nationwide by the various state programs [10]. While the number might be small, it should be noted that not all states require veterinarians to participate in PDMP thus it could be underreported [11]. The American Veterinary Medical Association (AVMA) also advocates the need for options for reporting of suspected opioid and controlled substance drug shoppers and/or diversion which could also impact the number of vet shoppers reported [12].

Recognizing the spread and persistence of the current opioid epidemic in Appalachia and the need for more information regarding vet shopping behavior, this study sought out to assess the attitudes, knowledge base and management practices among a cohort of veterinarians in South – South Central Appalachian states regarding vet shopping behavior. The data gathered from this study will provide a better understanding regarding the attitudes and practices used by veterinarians related to vet shopping and will provide insight for future research and intervention strategies regarding vet shopping.

## Results

Fourteen veterinarians from 14 different practices located within the Appalachian footprint of Kentucky, Tennessee, and Virginia participated in this qualitative study. Among the veterinarians interviewed, eight practiced in Tennessee, four in Kentucky, and two in Virginia. Table 1 displays practice characteristics of respondents (Questions 1–6, 10, and 12). Of the 14 veterinarians, 8 (57.1%) reported practicing medicine over 20, years. Thirteen veterinarians (92.9%) have heard of the practice of vet shopping. Eight veterinarians (57.1%) reported seeing 100 or above patients per week. The majority of veterinarians (9 [64.3%]) practiced in a clinic catering exclusively to small animals. Eight of 14 (57.1%) veterinarians reported personally encountering vet shopping in their own practice. Two veterinarians (14.3%) indicated vet shopping was not a problem. Four veterinarians (28.6%) reported receiving training on prevention and/or management of vet shopping. One veterinarian (7.1%) was found to be unaware of the phenomenon of vet shopping.

**Table 1:** Practice Characteristics of Respondents (*n* = 14)

Questions	Themes	Subthemes	***n*** (%)
1. How many years have you been in practice?	0–10		5 (35.6%)
	11–20		1 (7.1%)
	21–30		4 (28.6%)
	30–40		4 (28.6%)
2. Have you ever heard of the practice of vet shopping?	Yes		13 (92.9%)
	No		1 (7.1%)
3. How many new and returning patients do you see, on average, during a typical work week?	1–49		1 (7.1%)
	50–99		5 (35.6%)
	100–149		5 (35.6%)
	150–199		1 (7.1%)
	200+		2 (14.3%)
4. Please describe your practice structure (small animal predominant, mixed practice, large animal predominant).	Small-animal predominant		9 (64.3%)
	Large-animal predominant		1 (7.1%)
	Mixed-animal practice		4 (28.6%)
5. Have you ever encountered Vet shopping?	Yes		8 (57.1%)
	No		6 (42.9%)
6. To what extent do you think Vet Shopping is a problem in our Tri-State region?	Not a problem		2 (14.3%)
	It is a problem	Minor problem	4 (28.6%)
		Somewhat of a problem	7 (50%)
		A big problem	1 (7.1%)
10. Have you received any training on prevention and/or management of Vet Shopping?	Yes		4 (28.6%)
	No		10 (71.4%)
12. Is there information sharing between human doctors, veterinarians, and other entities such as law enforcement about current drug seeking behaviors or warnings about people seeking drugs (e.g. Break-ins at clinics)?	Yes		7 (50%)
	No		6 (42.9%)
	Uncertain		1 (7.1%)

Table 2 displays thematic analysis of qualitative data (Questions 7–9, 11, and 13) which highlights the six themes identified: interprofessional communication, knowing clients and patients, education, information sharing/database, judicious use of opioids, optimistic/not concerned. Tracking of verbalized responses were cumulatively totaled throughout all interviews. That is, an interviewee may have mentioned a particular theme twice, in response to two different questions, which would have counted as two verbalizations towards the total.

**Table 2:** Thematic Analysis of Qualitative Data (*n* = 14)

Questions	Themes	Subthemes	Supporting Quotes	***n*** (%)
7. What is your opinion about Vet Shopping?	Optimistic/Not concerned		“I have not had many incidences of it”	2 (14.3%)
8. How can veterinarians help in prevention of Vet Shopping?	Interprofessional Communication			2 (14.3%)
		Communicate with Pharmacies/Pharmacists	“I think being in closer contact sometimers with the pharmacist. They have been extremely helpful…”	1 (7.1%)
		Communicate with law enforcement	“I mean you can report those people to animal control officers or even the sheriff’s department or law enforcement…”	1 (7.1%)
	Knowing Clients and Patients		“I think what you have to do is to know your clientele…”	10 (71.4%)
	Information sharing/database		“The biggest thing would be if we had some sort of database…”	2 (14.3%)
	Judicious Use of Opioids		“Don’t just overprescribe pain medications…”	10 (71.4%)
a. Probe: In your opinion, what would be the best practice to prevent Vet Shopping?	Interprofessional Communication			2 (14.3%)
		Communicate with Pharmacies/Pharmacists	“Well one thing you can do is call most of the drugs into the pharmacy…”	2 (14.3%)
	Knowing Clients and Patients		“Probably just adhere to that animal client veterinary relationship before anything would be prescribed.”	8 (57.1%)
	Information Sharing/Database		“Honestly, some sort of database…”	1 (7.1%)
	Judicious Use of Opioids		“...I think just being careful and not routinely dispensing too much…”	6 (42.6))
b. What factors might limit your ability to prevent vet shopping?	Interprofessional Communication			2 (14.3%)
		Communicate with Other Veterinary Professionals	“I guess some of that is we’re not sure if they have been to other people, to other veterinarians or not…”	2 (14.3%)
	Knowing Clients and Patients		“I guess people lying…”	11 (78.6%)
	Education		“Definitely the education of the veterinarians coming out that it is you know a problem…”	2 (14.3%)
9. How can veterinarians help in control or management of vet shopping?	Interprofessional communication			4 (28.6%)
		Communicate with Pharmacies/Pharmacists	“To work more closely with the pharmacist…”	2 (14.3%)
		Communicate with Other Veterinary Professionals	“Well communicating with other practices in the area…”	2 (14.3%)
	Knowing Clients and Patients		“I guess it goes back to knowing the patient and knowing what the patient actually needs.”	8 (57.1%)
	Education		“Being more aware of what’s going on…”	2 (14.3%)
	Information Sharing/Database		“Maybe even there should be some kind of database…”	3 (21.4%)
	Judicious Use of Opioids		“Honestly I think we could be a little less inclined to reach for pain meds on every occasion”	6 (42.9%)
11. What resources do you need to prevent Vet shopping?	Interprofessional communication			3 (21.4%)
		Communicate with Pharmacies/Pharmacists	“I do think you know a close relationship with pharmacies…”	1 (7.1%)
		Communicate with Other Veterinary Professionals	“More of a freedom within the community, the medical community, and the veterinary community to discuss the topics…”	1 (7.1%)
		Communicate with Law Enforcement	“I think you have to have a relationship with law enforcement…”	1 (7.1%)
	Knowing Clients and Patients		“...a good relationship with your client.”	7 (50.0%)
	Education		“A CE (continuing education) course that would…”	7 (50.0%)
	Information Sharing/Database		“I think maybe the CASPR reports where we can see if an individual has a history of using a particular medication.”	1 (7.1%)
	Optimistic/Not Concerned		“I think we have the resources we need…”	1 (7.1%)
13. What are some things that you think veterinarians can do to help address the opioid crisis?	Knowing Clients and Patients		“Just know your clients, know their needs, and be sure you have a doctor-client-patient relationship”	6 (42.9%)
	Judicious Use of Opioids		“And to be sparing in your prescriptions as far as in the amount and number of days…”	11 (78.6%)

### Interprofessional communication

Veterinarians interviewed in this study expressed a desire to communicate generally and more efficiently with other veterinary colleagues and medical professionals. The desire for the ability to communicate with pharmacies/pharmacists was verbalized six times throughout the interviews, expressing this value when questioned regarding methods to prevent vet shopping, best practices to prevent vet shopping, control/management of vet shopping, and resources needed to prevent vet shopping. The ability to communicate with other veterinary professionals was verbalized by five times during the course of the interviews with veterinarians conveying this value when questioned regarding limitations in their ability to prevent vet shopping, control/management of vet shopping and resources needed to prevent the practice of vet shopping.

### Knowing clients and patients

A valid veterinary-client-patient-relationship (VCPR) was a value expressed by multiple veterinarians within this study. VCPR was cumulatively mentioned 50 times throughout the course of the interviews. Interviewed veterinarians verbalized verbatim a valid VCPR or the tenets thereof when asked about methods to help in the prevention of vet shopping, best practices to prevent vet shopping, limitations in their ability to prevent vet shopping, control/management of vet shopping, resources needed to prevent vet shopping and how veterinarians could address the opioid crisis.

### Education

Veterinarians expressed the desire for more education for themselves and/or their staff members 11 times, verbalizing a desire for education when questioned regarding best practice for prevention of vet shopping, limitations in their ability to prevent vet shopping, control/management of vet shopping and resources needed to prevent vet shopping.

### Information sharing/database

Veterinarians expressed a need or desire to be able to track and control animal prescriptions through databases like the Kentucky All Schedule Prescription Electronic Reporting (KASPER). It was verbalized seven times when questioned regarding prevention and control of vet shopping and resources needed.

### Judicious use of opioids

Veterinarians emphasized the judicious use of opioid prescriptions, verbalizing it 33 times throughout the interviews. The judicious use of opioids was mentioned when asked about vet shopping prevention, control or management of vet shopping and addressing the opioid crisis.

### Optimistic/not concerned

Three of 14 veterinarians expressed optimism or non-concern regarding vet shopping.

## Discussion

The findings of this study show that veterinarians practicing in the tri-state region expressed a need for more efficient communication between themselves, other veterinary colleagues, medical professionals including pharmacists and law enforcement regarding the use of prescription opioids by clients and their pets. Only two out of the 14 veterinarians interviewed believed that vet shopping was not a concern for them. The remaining 12 believed it to be a problem that needed to be addressed. One potential solution to improve communication and gain information concerning prescription opioid use by clients and their pets is through a prescription drug monitoring program.

While laws are currently in place across the nation to curb human physician shopping, only 20 states and District of Columbia have addressed the vet shopping issue by requiring veterinarians to report dispensing of controlled substances into Prescription Drug Monitoring Programs (PDMPs) [11]. PDMP programs have historically proven successful in limiting human physician shopping. For example, the CDC reports that in 2012, after a law requiring human medical prescribers to check the state’s PDMP before prescribing opioids was put into place, there was a 75% drop in doctor shopping the following year [13].

Veterinarians may be opposed to the restrictions placed on them by new legislation, just as many human medical doctors likely pushed back against an addition to their already busy workflow when PDMP programs were implemented in human medicine. But because it is a possible avenue for addicts and drug dealers to obtain opioids, it must be addressed. This study showed veterinary professionals express a desire to be able to track animal prescriptions through databases and learn more and take action regarding this epidemic. Through PDMPs and education, veterinarians can be more vigilant of this issue and prevent owners from dishonestly obtaining prescriptions.

One of the greatest tools to aid in the accurate identification of a drug seeking client is a relationship with the client and patient. Simply stated, veterinarians are able to identify patients who seek drugs, if they know them. There is no magical way to form relationships with clients who are first timers, and although relationships are built over time, there are certain red flags to watch out for when treating patients.

Signs of an owner seeing a veterinarian simply for controlled substances may be subtle but recognizable if a veterinarian watches for them. Red flags include: new patients bringing in seriously injured animals, owners describing symptoms that are inconsistent with the exam or which require specific medications, requests for medications by name, requesting refills, or aggressive behavior [8]. Other warning signs include: pets with chronic injuries, new injuries, and injuries with unknown causes [14]. While this paper provides actionable information, more research should be done to better understand the challenges veterinarians face when confronted with the aforementioned red flag scenarios and more explicit guidelines should be sought to aid clinicians in these challenging, and potentially more frequent, situations.

As a recurring theme throughout the interviews was the desire for more veterinarian education on this topic, the aforementioned information regarding ‘red flags’ and other related knowledge must be better disseminated among the profession in order to quell illicit opioid access. One method used to address public health concerns is by educating practitioners through continuing education programs. Multiple states in the US have begun to require pain management/controlled substance prescribing continuing education (CE) requirements for human physicians [15]. Opioid and other controlled substance prescribing CE programs may be an effective avenue to improve veterinarian education regarding vet shopping. As this study found, judicious use of opioids was a primary concern among veterinarians and potential point of improvement in addressing vet shopping and the opioid crisis as a whole. Continuing education programs could encourage the judicious use of opioids by veterinarians and prove an actionable and effective step towards addressing this issue.

However, there are several limitations in this study. One potential limitation is a bias of convenience in that the veterinarians were recruited using an online search engine. Thus, veterinarians who did not have an online presence were excluded from the study. Recruitment of participants was also subject to geographical bias, as veterinarians involved in practicing only in the Central – South Central Appalachian states of Tennessee, Virginia, and Kentucky were included in this study. Furthermore, the method of data collection was through telephone interviews which does not allow for participant observation. Future studies can incorporate face-to-face interviews. While this was a qualitative study, the sample size could certainly have been more diverse. Due to the anonymous nature of the survey it was a challenge to include certain descriptive data. Future studies could include more descriptive data, such as gender and specific geographical data of veterinarians interviewed in order to better characterize the respondents in the region studied. The sample size consisted of 14 participants which limits the conclusions drawn from this study - future studies with a more robust sample would be of value.

Taking data from the Association of American Veterinary Medical Colleges (AAVMC) and DATAUSA, we can estimate the mean number of years practiced for all veterinarians to be 17.3 [16, 17]. Our results show that the mean number of years of surveyed participants practicing veterinary medicine was 21. Thus, the veterinarians surveyed may be older and have likely practiced longer than the average U.S. veterinarian. This age and practice experience discrepancy may have influenced perceptions of the opioid crisis. Most of the respondents were small animal clinicians; however, no studies to our knowledge associate vet shopping frequency and small animal practices. Regardless, this may impact generalization of this study’s findings and further studies should be conducted to investigate if this phenomenon occurs more frequently in small animal practices.

There were also some limitations regarding the data analysis. The psychometric properties of the instrument developed by the researchers, beyond face and content validation, were not established. Future studies may address establishing internal consistency reliability, test-retest reliability, and construct validation. Triangulation with elaborate quantitative methods was not done in this study. Future studies must utilize systematic mixed-methods designs to enhance the creditworthiness and trustworthiness of the results. Qualitative statistical software such as N-Vivo or Atlas were not used and manual thematic analysis was done, which can add to greater subjectivity.

## Conclusions

Appreciating the extent of the opioid epidemic in Appalachia and the gap in information regarding vet shopping behavior, this study sought to assess the attitudes, knowledge base and management practices amongst a cohort of veterinarians in South – South Central Appalachia regarding vet shopping. The overwhelming majority of veterinarians’ responses highlighted the importance of information sharing/databases, interprofessional communication, continuing education and advocating the judicious use of opioids in addressing this insidious phenomenon. These results suggest a need for a nationwide veterinarian accessible prescription drug monitoring program and the need for more research to address gaps in current state PDMP reporting versus what is encountered in veterinary practice, as many of the practices encountered vet shopping, but very few reports of vet shopping were found in a nationwide search. The results of this study also indicate that continuing education programs highlighting pain management/controlled substance prescribing, the judicious use of opioids, and red flag drug seeking behavior, amongst others, could be beneficial in quelling vet shopping. As a qualitative study with limited sample size, prudence must be taken with these generalizations and serve as an impetus for future study and justification for deeper investigation into the unfortunate practice of opioid solicitation via vet shopping.

## Methods

### Participants and procedure

A list of potential participants was generated using a directory of LMU College of Veterinary Medicine clinical affiliates supplemented with an internet search of veterinarians in the Appalachian Region of Kentucky, Tennessee, and Virginia. The survey instrument (Additional file 1: Vet Shopping Survey) utilized was developed by researchers of this study. Potential participants were then contacted via phone call [Institutional Review Board (IRB); Protocol #:705 V.1]. First contact was made to a receptionist at the clinic, who then gave us contact to the veterinarian. A script was read at the start of the interview to relay the purpose of the interview and to explain the research study. The script also explained that the interview would be audio recorded, and oral consent for participation in the study was obtained. Interviews with veterinarians were conducted via speakerphone and recorded using Olympus V414111 SU000 Digital Dm-720 Voice Recorder. No identifiable markers were collected during the interview process and confidentiality/anonymity of subjects was maintained. Thirteen questions were asked during the interview plus a few probes. Any summaries of interviews were sent to veterinarians via email upon request. The audio recording was then transferred to another researcher for transcription.

### Instrument

At the start of the interview, the study was explained followed by a 13-question interview with several probes assessing veterinary attitudes and practices regarding vet shopping. Questions 1, 3, and 4 inquired about the veterinarian’s practice, including how many years they have been in practice, the number of patients seen per week, and their practice structure (small animal predominant, mixed practice, or large animal predominant). Questions 2 and 5 assessed whether or not they had ever heard of or encountered vet shopping. Questions 6 and 7 asked their opinion about vet shopping and the extent to which vet shopping was a problem in the tri-state region. Questions 8 and 9 assessed how veterinarians can help in the prevention or control of vet shopping. Furthermore, probing questions for 8 and 9 assessed what the best practices and limiting factors were to prevent or control vet shopping. Questions 10–12 asked if the veterinarian had received any training on prevention/management of vet shopping, any resources they need to prevent vet shopping, and whether there was any information sharing between human doctors, veterinarians and other entities such as law enforcement about current drug seeking behaviors. Finally, question 13 inquired about possible things vets could do to help address the opioid crisis.

### Data analysis

All audio recordings were fully transcribed. Responses from the interview were taken from the transcripts and organized by question on a spreadsheet. The data were analyzed using a thematic analysis approach [18]. Initially, researchers created a preliminary list of codes from the responses for each question. The researchers then collaborated to make a combined, agreed-upon list of codes. Afterwards, the researchers worked together to organize the codes into broader categories grouped based on similarities. These were then grouped into themes. A thematic analysis table was made using each question and response, highlighting the number of responses corresponding to each theme, including short quote examples. Descriptive statistics were carried out with the responses to each item using Statistical Package for the Social Sciences (SPSS) version 25.

## Supplementary information

**Additional file 1.** Vet Shopping Survey.

## Data Availability

The datasets used and/or analyzed during the current study are available from the corresponding author on reasonable request.

## Abbreviations

AVMA: American Veterinary Medical Association
CDC: Centers for Disease Control and Prevention
CE: Continuing Education
IRB: Institutional Review Board
KASPER: Kentucky All Schedule Prescription Electronic Reporting
PDMP: Prescription Drug Monitoring Programs
SPSS: Statistical Package for the Social Sciences
US: United States
VCPR: Veterinarian-Client-Patient-Relationship
