# Supplementary material for: Veterinarians’ attitudes and practices regarding opioid-related vet shopping practices in tri-state Appalachian counties: an exploratory study
Source: BMC Vet Res. 2020 Jun 22;16:210. doi: 10.1186/s12917-020-02428-x (PMC7310014; doi:10.1186/s12917-020-02428-x)
Supplement: Supplementary file 1 — Additional file 1. Vet Shopping Survey. [file 12917_2020_2428_MOESM1_ESM.docx]

**Vet Shopping Survey**

Hello. I am ____________. I am a student working with the Center for Animal and Human Health in Appalachia at Lincoln Memorial University working under the supervision of Dr. Gilbert Patterson, Assistant Professor of Veterinary Medicine. You have been identified as a veterinarian practicing in Tri-state Appalachian counties.

We are interviewing veterinarians to assess their attitudes and practices regarding vet shopping (i.e., a practice in which people will maim or injure their own pets to obtain drugs from veterinarians to feed their own addictions). Completing the interview is voluntary and will not change the relationship with LMU if not completed. The interview will be audio recorded and destroyed 3 years after the completion of the study as mandated by federal law. This project has received LMU Institutional Review Board approval. Your responses will help design better ways to inform veterinarians how to recognize, prevent, and manage vet shopping. Your name will be kept confidential, and any other identifying markers will be destroyed.

Would you be willing to participate in this study? It will probably take no more than 20 minutes of your valuable time.

Participant: “Yes” or “No” If “NO” 🡪 Thank you for your time.

If “YES” 🡪 We will be asking you a set of 13 questions plus any probes to assess your attitudes and practices regarding vet shopping. Do I have your kind permission to proceed?

1. How many years have you been in practice? (Interviewer: approximate number of years will do)

2. Have you ever heard of the practice of vet shopping?

3. How many new and returning patients do you see, on average, during a typical work week?

4. Please describe your practice structure (small animal predominant, mixed practice, large animal predominant)

5. Have you ever encountered Vet Shopping?

6. To what extent do you think Vet Shopping is a problem in our Tri-State region?

7. What is your opinion about Vet Shopping?

8. How can veterinarians help in prevention of Vet Shopping?

a. Probe: In your opinion, what would be the best practice to prevent Vet Shopping?

b. Probe: What factors might limit your ability to prevent Vet Shopping?

9. How can veterinarians help in control or management of Vet Shopping?

a. Probe: In your opinion, what would be the best practice to control Vet Shopping?

b. Probe: What factors might limit your ability to control Vet Shopping?

10. Have you received any training on prevention and/or management of Vet Shopping?

11. What resources do you need to prevent Vet Shopping?

12. Is there information sharing between human doctors, veterinarians, and other entities such as law enforcement about current drug seeking behaviors or warnings about people seeking drugs (e.g. Break-ins at clinics)?

13. What are some things that you think veterinarians can do to help address the opioid crisis?

Thank you very much for your time. Do you need summary results of the report? If yes, Please provide your e-mail address.
